# New endoscopic finding of esophageal achalasia with ST Hood short type: Corona appearance

**DOI:** 10.1371/journal.pone.0199955

**Published:** 2018-07-31

**Authors:** Hironari Shiwaku, Kanefumi Yamashita, Toshihiro Ohmiya, Satoshi Nimura, Yoshiyuki Shiwaku, Haruhiro Inoue, Suguru Hasegawa

**Affiliations:** 1 Department of Gastroenterological Surgery, Faculty of Medicine, Fukuoka University, Fukuoka, Japan; 2 Department of Pathology, Faculty of Medicine, Fukuoka University, Fukuoka, Japan; 3 Crystal Building Clinic, Fukuoka, Japan; 4 Digestive Disease Center, Showa University Koto Toyosu Hospital, Tokyo, Japan; University Hospital Llandough, UNITED KINGDOM

## Abstract

**Background and study aims:**

Detecting esophageal achalasia remains a challenge. We describe the diagnostic utility of corona appearance, a novel endoscopic finding specific to esophageal achalasia.

**Patients and methods:**

Corona appearance and seven conventional endoscopic findings were compared for sensitivity and consistency (κ-value) among 53 untreated esophageal achalasia patients who underwent endoscopy at our hospital. The following criteria had to be met during lower esophageal sphincter examination using the attached ST Hood short-type for positive corona appearance: A) congestion inside the hood, B) ischemic change around the hood, and C) palisade vessels outside the hood.

**Results:**

Corona appearance had the highest sensitivity (91%; κ-value, 0.71). Other findings in descending order of sensitivity included 1) functional stenosis of the esophagogastric junction (EGJ; 86%; κ-value, 0.58), 2) mucosal thickening and whitish change (71%; κ-value, 0.27), 3) abnormal contraction of the esophageal body (59%; κ-value, 0.32), 4) dilation of the esophageal lumen (58%; κ-value, 0.53), 5) liquid remnant (57%; κ-value, 0.51), 6) Wrapping around EGJ (49%; κ-value, 0.14), and 7) food remnant (30%; κ-value, 0.88). Even in 22 patients with poor (grade 1) intraluminal expansion, corona appearance had highest sensitivity (88%) compared to other endoscopic findings (κ-value, 0.63).

**Conclusions:**

Among endoscopic findings using a ST Hood short-type to diagnose esophageal achalasia, corona appearance had the highest sensitivity and its consistency (κ-value) among endoscopists was substantial compared to other endoscopic findings. Similar results were obtained for esophageal achalasia cases with poor expansion. Endoscopic diagnosis of esophageal achalasia with hood attached is useful.

## Introduction

Esophageal achalasia is a motility disorder of the esophagus, primarily involving relaxation failure of the lower esophageal sphincter (LES) and abnormal contraction of the esophagus body [1]. Until the advent of peroral endoscopic myotomy (POEM), conventional treatment methods for esophageal achalasia included endoscopic balloon dilation and surgery [2–4]. However, POEM, devised by Inoue *et al*. in 2008, is a procedure by which the treatment of esophageal achalasia was further developed [5]. It is now performed worldwide as an epoch-making treatment of esophageal motility disorders, including esophageal achalasia [6–11]. Although POEM is currently being established as a s1 treatment method for esophageal achalasia, the extent to which esophageal achalasia can be detected remains an important issue. Esophageal manometry is currently the most effective diagnostic method [12, 13]; however, not all institutions have the requisite means to perform it. Furthermore, esophageal manometry is associated with pain, and therefore, it is not appropriate for screening tests. Conversely, endoscopy is a test that is extensively used, which can minimize the pain associated with the examination if performed under intravenous anesthesia. If a diagnostic method could be established for esophageal achalasia with high endoscopic sensitivity, then more esophageal achalasia patients could be identified with less pain. In the present study, we introduce an endoscopy-based diagnostic method with high sensitivity “Corona appearance” using an ST Hood short-type [14].

## Materials and methods

### Patients

Subjects included 78 achalasia patients who underwent POEM at our institution between March 2014 and December 2015. Esophageal achalasia was diagnosed by esophagogastroduodenoscopy, barium esophagogram, computed tomography (CT), and manometry. Patient details are shown in Table 1. Twenty-five patients who had previously received balloon dilation or surgical myotomy were excluded, and the remaining 53 untreated patients were retrospectively evaluated. This study also included a control group (n = 53; 45 healthy subjects, 5 cases of reflux esophagitis, and 3 cases of eosinophilic esophagitis).

**Table 1:** General clinical features of patients with achalasia.

Gender, male:female	23:30
Age, mean (range)	48 (15–83) years
Duration of disease, mean (range)	110 (3–504) months
Disease type, straight:sigmoid	45:8
Degree of dilation, I:II:III	22:28:3
LES pressure (range)	51 (16–212) mmHg

LES: lower esophageal sphincter

This study was approved by the Ethics committee of Faculty of Medicine, Fukuoka University (approval number: 2016M052). Written informed consent was obtained from all patients and the possibility of using hood was mentioned in the consent form before examination. The study adhered to the tenets of the Declaration of Helsinki.

### Endoscopic examination

All patients underwent endoscopy under anesthesia (midazolam/pethidine hydrochloride) without the use of anticholinergic drugs. A GIF-H260 scope was used with an ST Hood short-type (DH-28GR; Fujifilm, Japan) attached in the first esophageal observation [14]. After the endoscope was removed, observations were performed again without the ST Hood short-type.

### Conventional endoscopic features

**Fig 1:**
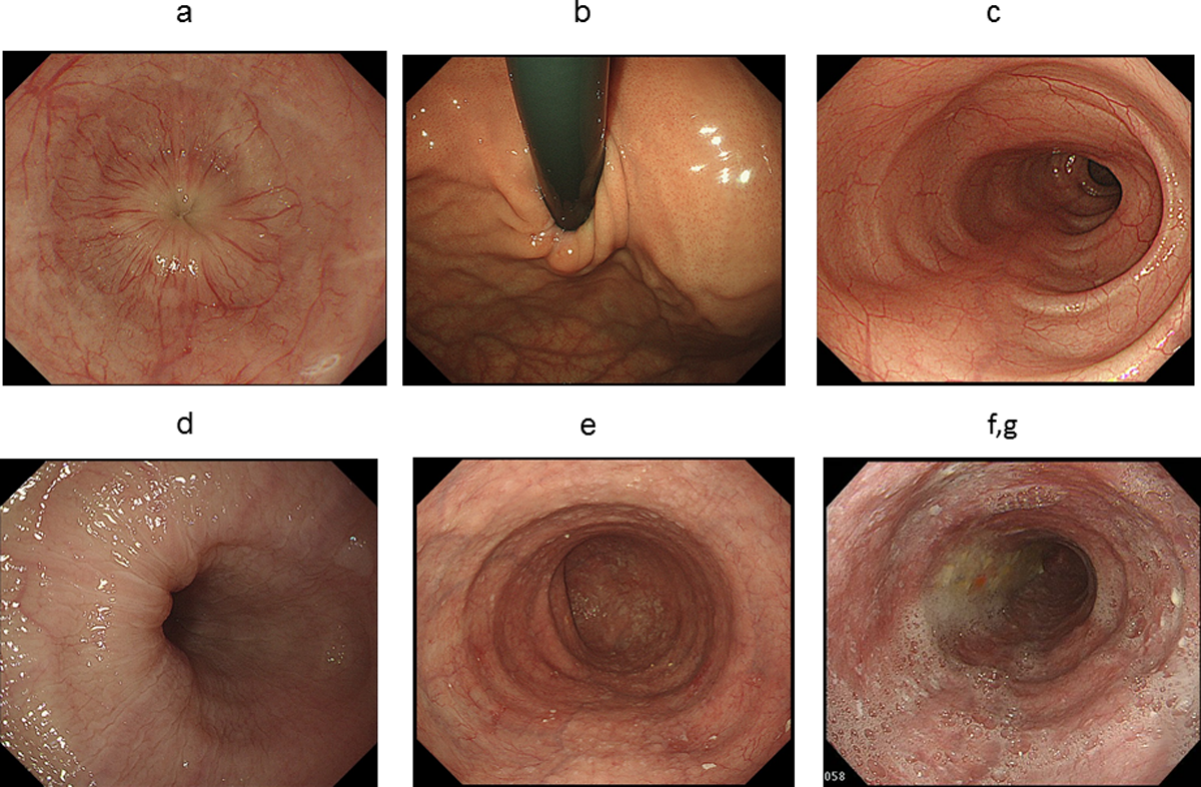
Endoscopic findings in esophageal achalasia. a: Functional stenosis of the esophagogastric junction. b: Wrapping around the esophagogastric junction. c: Abnormal contraction of the esophageal body. d: Mucosal thickening and whitish change. e: Dilation of the esophageal lumen. f, g: Liquid and/or food remnant.

The endoscopic findings evaluated were those described in the Japanese guidelines for esophageal achalasia (2012; Fig 1) [15] and included the following:

(a) functional stenosis of the esophagogastric junction (EGJ), (b) wrapping around EGJ, (c) abnormal contraction of the esophageal body, (d) mucosal thickening and whitish change, (e) dilation of the esophageal lumen, (f) liquid remnant, and (g) food remnant.

In the guidelines, (a) and (b) and (f) and (g) are merged. However, in this study, we evaluated each finding to be as accurate as possible.

### Corona appearance

**Fig 2:**
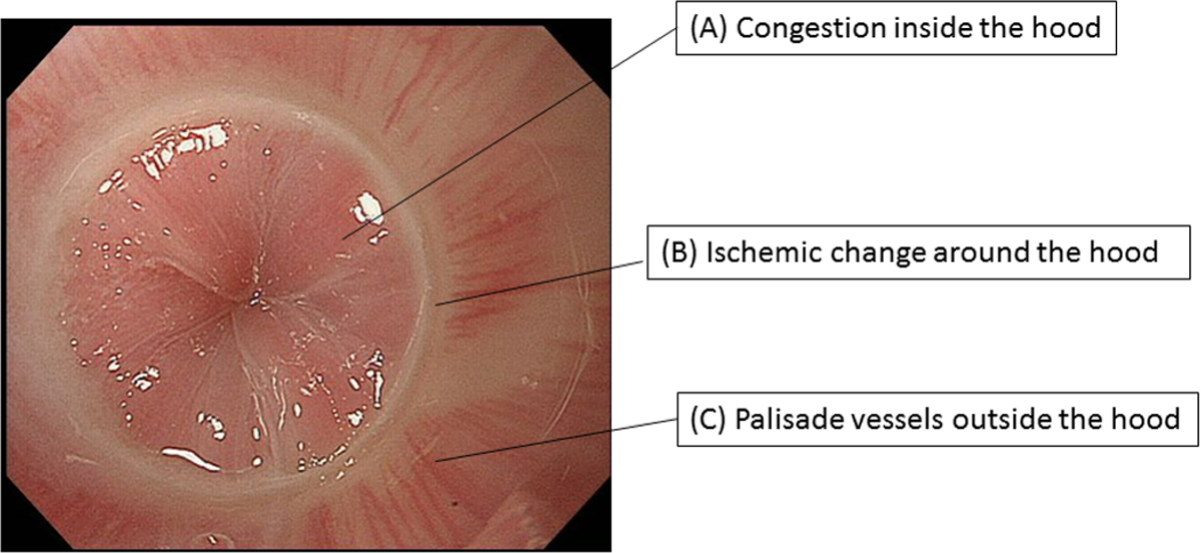
CA was defined as positive when all of the following three criteria were met upon observation of the LES performed with the attached ST Hood short-type: a) Congestion inside the hood, b) ischemic change around the hood, and c) palisade vessels outside the hood.

Corona appearance (CA) was defined as positive when all of the following three criteria were met upon observation of the LES performed with the attached ST hood short-type: (a) congestion inside the hood, (b) ischemic change around the hood, and (c) palisade vessels outside the hood (Fig 2, Video).

### Evaluation of endoscopic features

Evaluations were performed by three endoscopists, including S with more than 10 years of experience, Y with more than 5 years and less than 10 years, and O with less than 5 years of experience. O was a beginner for the endoscope and his experience of upper endoscopic examination was less than 100 cases. Irrespective of whether the hood was attached or not, the endoscopic findings that could be observed with the highest clarity were given priority and used in the evaluations.

### Statistical analysis

The inter-observer agreement levels were analyzed in eight categories using Fleiss’ multiple-rater Kappa analysis. A general consensus scheme for strength of agreement by κ-value was used in the evaluation as follows: 0.2–0.4, fair; 0.4–0.6, moderate; 0.6–0.8, substantial; and 0.8–1, excellent.

## Results

Among the endoscopic findings, CA had the highest sensitivity (91%) and a κ-value of 0.71. Other findings in descending order of sensitivity included 1) functional stenosis of EGJ (sensitivity 86%, κ-value 0.58), 2) mucosal thickening and whitish change (sensitivity 71%, κ-value 0.27), 3) abnormal contraction of the esophageal body (sensitivity 59%, κ-value 0.32), 4) dilation of the esophageal lumen (sensitivity 58%, κ-value 0.53), 5) liquid remnant (sensitivity 57%, κ-value 0.51), 6) wrapping around EGJ (sensitivity 49%, κ-value 0.14), and 7) food remnant (sensitivity 30%, κ-value 0.88) (Table 2). Furthermore, on examination of patients with poor intraluminal dilatation (grade I dilatation, 22 patients), we found a CA sensitivity of 88% with a κ-value of 0.63, yielding the highest sensitivity of all the endoscopic findings (Tables 3 and 4). The CA was only observed in patients with achalasia. None of the non-achalasia patients showed positivity for CA, including the cases of reflux esophagitis and eosinophilic esophagitis.

**Table 2:** General sensitivity and κ-value of endoscopic findings in achalasia.

	Sensitivity (range) (%)	*k* value
Corona appearance	91 (87–96)	0.71
Functional stenosis of EGJ	86 (81–89)	0.58
Mucosal thickening and whitish change	71 (64–79)	0.27
Abnormal contraction of the esophageal body	59 (36–72)	0.32
Dilation of the esophageal lumen	58 (53–66)	0.53
Liquid remnant	57 (43–68)	0.51
Wrapping around EGJ	49 (23–77)	0.14
Food remnant	30 (26–32)	0.88

EGJ: esophagogastric junction

**Table 3:** Clinical features of patients with undilated achalasia (degree of dilation I).

Gender, male:female	7:15
Age, mean (range)	53 (18–82) years
Duration of disease, mean (range)	94 (3–504) months
LES pressure (range)	51 (16–118) mmHg

LES: lower esophageal sphincter

**Table 4:** Sensitivity and κ-value of endoscopic findings in undilated achalasia (degree of dilation I).

	Sensitivity (Range) (%)	*k* value
Corona appearance	88 (82–95)	0.63
Functional stenosis of EGJ	82 (77–86)	0.59
Mucosal thickening and whitish change	55 (45–68)	0.13
Abnormal contraction of the esophageal body	49 (27–59)	0.45
Wrapping around EGJ	38 (14–59)	0.05
Liquid remnant	30 (14–41)	0.51
Dilation of the esophageal lumen	27 (23–36)	0.44
Food remnant	9 (9)	1.00

EGJ: esophagogastric junction

## Discussion

Esophageal achalasia is a motility disorder of the esophagus that primarily involves relaxation failure of the LES and abnormal contraction of the esophagus body [1]. Before the introduction of POEM, conventional treatment methods for esophageal achalasia mainly included endoscopic balloon dilation and surgery [2–4]. POEM is a breakthrough treatment devised by Inoue *et al*. in 2008, who expanded on Heller myotomy to include endoscopic treatment, and can therefore be regarded as “Heller myotomy without injuring the body surface” [5]. POEM is currently being established as a stable treatment method for esophageal achalasia; however, the extent to which esophageal achalasia can be detected is an important clinical task in future.

In the diagnosis of esophageal achalasia, it is of highest importance to check for achalasia on the basis of symptoms (dysphagia, regurgitation, chest pain, and weight loss) [16]. In the event that achalasia is suspected on the basis of symptoms, tests such as endoscopy, contrast esophagography, esophageal manometry, and CT should be considered. For the diagnosis of achalasia, esophageal manometry is the most reliable test; however, a hard large catheter is inserted through the nose into the stomach in the conscious state, making the test very painful. Furthermore, although contrast esophagography enables objective evaluation and is thus a useful test, endoscopy is the first test performed in an actual medical setting.

Among the endoscopic findings included in the esophageal achalasia treatment guidelines, functional stenosis of the esophagogastric junction and abnormal contraction of the esophageal body are the findings that reflect the actual state of achalasia, with all other findings being secondary. Furthermore, with regard to abnormal contraction of the esophageal body, it is challenging to objectively evaluate such an abnormality using endoscopy alone.

Therefore, detecting functional stenosis of the esophagogastric junction is very important while diagnosing achalasia endoscopically. Iwakiri et al. reported that rosette-like esophageal folds were observed in the lower esophagus after deep inspiration appeared in 33/34 achalasia patients [17]. This endoscopic finding has high sensitivity but requires a fully conscious state.

The novel method introduced in the present study using a hood can visualize functional stenosis of this lesion more clearly and objectively with high sensitivity and concordance rate. Furthermore, this method can be performed under intravenous anesthesia.

**Fig 3:**
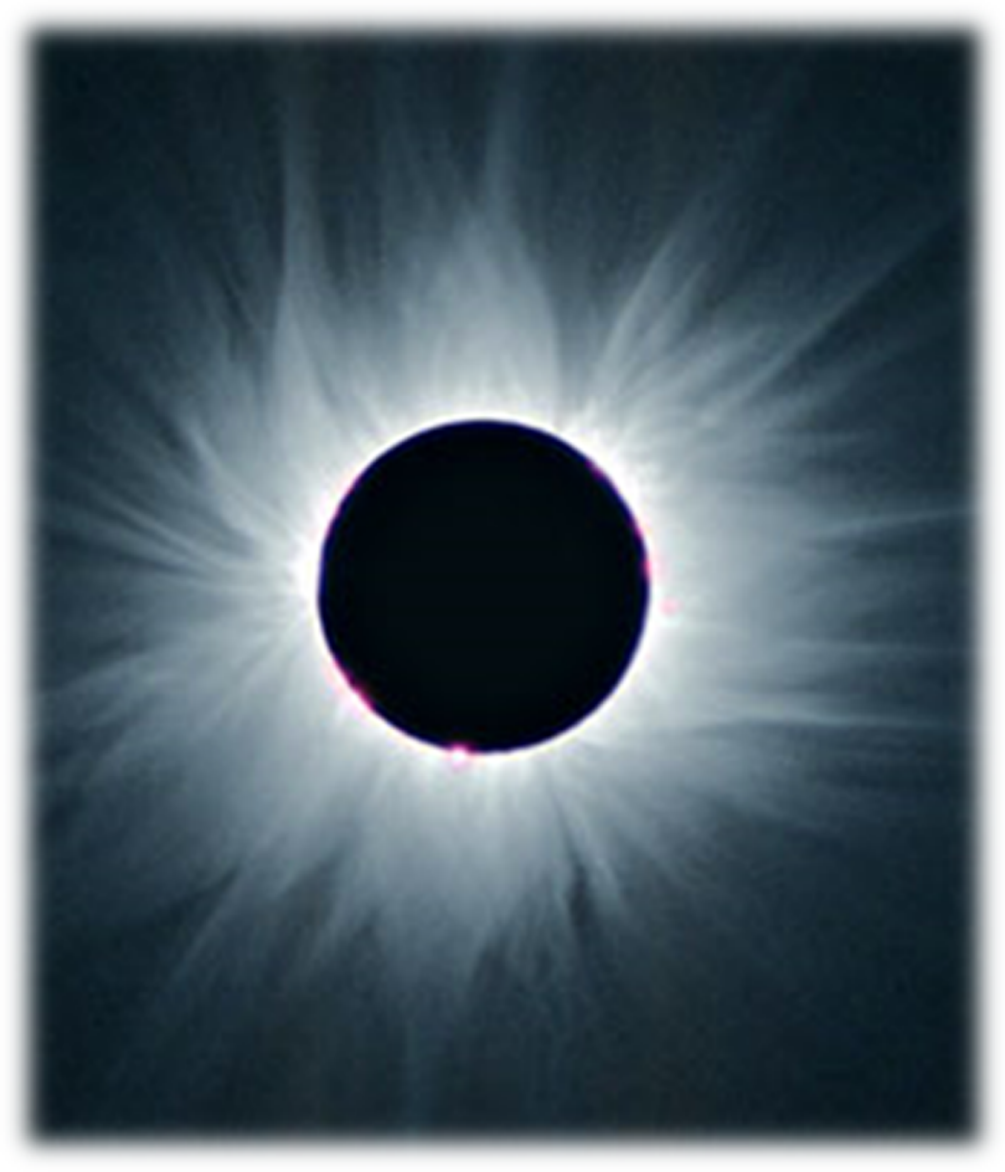
Corona of the sun. Source of photo: Nisshoku no subete, chasing shadows, an observer’s guide to solar eclipses, Osamu Ohgoe, Kazuo Shiota, Seibundo Shinkosha Publishing Co., Ltd. Quoted by the permission of Seibundo Shinkosha Publishing Co., Ltd. and Kazuo Shiota provided permission to republish this image under the Creative Commons Attribution (CC BY) 4.0 license.

With the attached ST Hood short-type, congestion inside the hood, ischemic change around the hood, and palisade vessels outside the hood can be observed consistently in LES in achalasia patients. We named these findings as CA because of the resemblance to the sun’s corona observed in the event of a total eclipse and examined its diagnostic efficacy (Fig 3). Among the endoscopic findings, CA had the highest sensitivity (91%) and a κ-value of 0.71. Among the current endoscopic findings using the ST Hood short-type, CA had the highest sensitivity with regard to endoscopic diagnosis of esophageal achalasia with a substantial concordance rate.

These results therefore suggest that when endoscopically diagnosing esophageal achalasia, observation with an attached ST Hood short-type may be useful for detecting esophageal achalasia. In the actual clinical setting, it is relatively easy to diagnose achalasia with the esophageal diameter expanded. However, it is difficult to diagnose achalasia with poor dilatation by tests, particularly with endoscopy, other than esophageal manometry.

In the present study, even when examining patients with poor intraluminal dilatation (grade I dilatation, 22 patients), we found a CA sensitivity of 88% with a κ-value of 0.63, revealing the highest sensitivity compared with other endoscopic findings. In addition, it appeared that CA is useful for detecting achalasia with poor dilatation.

**Fig 4:**
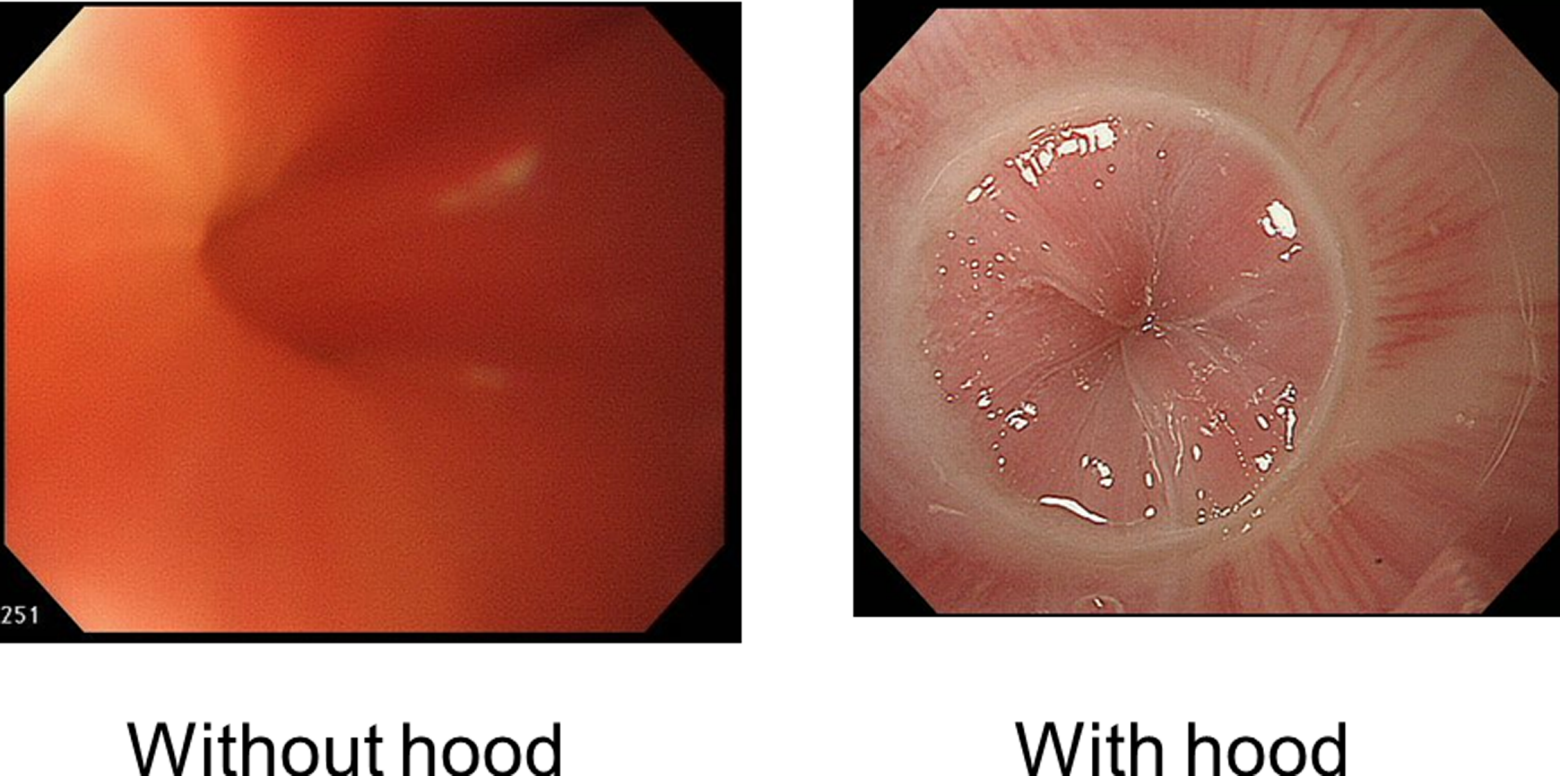
Endoscopic findings of the LES with hood (right) and without hood (left).

Unlike esophageal manometry, endoscopy can be performed at any institution. Therefore, institutions that are unable to perform esophageal manometry can perform endoscopy with an ST Hood short-type attached, thus enabling them to detect esophageal achalasia with high sensitivity. Furthermore, the use of a hood enables more detailed observation of the LES, and thus, the presence or absence of comorbidity can be accurately ascertained (Fig 4).

The present study was limited by its retrospective nature and the fact that the study was performed at a single institution with a small population. In future, a prospective study that includes more subjects and considers other endoscopic findings for esophageal achalasia is warranted[18].
